# Development of prediction equations for estimating appendicular skeletal muscle mass in Japanese men and women

**DOI:** 10.1186/s40101-017-0150-x

**Published:** 2017-08-29

**Authors:** Taishi Furushima, Motohiko Miyachi, Motoyuki Iemitsu, Haruka Murakami, Hiroshi Kawano, Yuko Gando, Ryoko Kawakami, Kiyoshi Sanada

**Affiliations:** 1 College of Sport and Health Science, Ritsumeikan University, 1-1-1 Noji Higashi, Kusatsu, Shiga 525-8577 Japan; 2 Department of Health Promotion and Exercise, National Institute of Health and Nutrition, 1-23-1 Toyama, Shinjuku-ku, Tokyo, 162-8636 Japan; 3 Faculty of Letters, Kokushikan University, 4-28-1 Setagaya, Setagaya-ku, Tokyo, 154-8515 Japan; 4 Faculty of Sport Sciences, Waseda University, 2-579-15 Mikajima, Tokorozawa, Saitama 359-1192 Japan

**Keywords:** Sarcopenia, Appendicular skeletal muscle, Prediction equation, Cardiovascular disease, Osteoporosis

## Abstract

**Background:**

This study aimed to develop and cross-validate prediction equations for estimating appendicular skeletal muscle mass (ASM) and to examine the relationship between sarcopenia defined by the prediction equations and risk factors for cardiovascular diseases (CVD) or osteoporosis in Japanese men and women.

**Methods:**

Subjects were healthy men and women aged 20–90 years, who were randomly allocated to the following two groups: the development group (D group; 257 men, 913 women) and the cross-validation group (V group; 119 men, 112 women). To develop prediction equations, stepwise multiple regression analyses were performed on data obtained from the D group, using ASM measured by dual-energy X-ray absorptiometry (DXA) as a dependent variable and five easily obtainable measures (age, height, weight, waist circumference, and handgrip strength) as independent variables.

**Results:**

When the prediction equations for ASM estimation were applied to the V group, a significant correlation was found between DXA-measured ASM and predicted ASM in both men and women (*R* ^2^ = 0.81 and *R* ^2^ = 0.72). Our prediction equations had higher *R* ^2^ values compared to previously developed equations (*R* ^2^ = 0.75–0.59 and *R* ^2^ = 0.69–0.40) in both men and women. Moreover, sarcopenia defined by predicted ASM was related to risk factors for osteoporosis and CVD, as well as sarcopenia defined by DXA-measured ASM.

**Conclusions:**

In this study, novel prediction equations were developed and cross-validated in Japanese men and women. Our analyses validated the clinical significance of these prediction equations and showed that previously reported equations were not applicable in a Japanese population.

## Background

Skeletal muscle is an important component of body composition and a key metabolic tissue related to physical function and health status. Sarcopenia, a geriatric syndrome characterized by reduced muscle mass and function [1], leads to physical disabilities [2–4], falls [4], and osteoporosis [5, 6]. It also increases the risk of chronic diseases, including cardiovascular diseases (CVD) [7, 8] and type 2 diabetes [9]. The prevalence of type 2 diabetes is increasing more rapidly among Asians compared to Caucasians, despite the fact that Asians have an overall lower BMI [10]. We previously reported that although sarcopenia is associated with thin body mass, it is associated with more glycation of serum proteins in Japanese adult men, independently of waist circumference [7]. Previous studies also have been reported that sarcopenia is associated with mortality [11], falls [12], impaired activities of daily living [13], and cognitive deterioration [14] in older Japanese adults. Many studies have used appendicular skeletal muscle mass (ASM) to define sarcopenia. Therefore, accurate and practical measurement of ASM is important for assessing and diagnosing sarcopenia in clinical settings.

There are several methods available for measuring skeletal muscle mass, including quantitative techniques such as magnetic resonance imaging and computed tomography, which show excellent accuracy [15]. However, the use of these techniques is limited in research and clinical practice due to their high cost, lack of portability, and risk of radiation exposure. Alternatively, dual-energy X-ray absorptiometry (DXA) can be used to estimate skeletal muscle mass with high accuracy and less radiation exposure compared to other imaging modalities. Although DXA-measured ASM has been used widely to diagnose sarcopenia [4, 7, 16–18], DXA is not portable and thus impractical in large field-based studies and epidemiologic studies. Thus, there is a need for a simple, valid, reliable, innocuous, and inexpensive method for measuring skeletal muscle mass.

Anthropometry could offer a practical alternative in estimating skeletal muscle mass. While several prediction models have been developed to estimate ASM [4, 19–23], no study has examined the relationship between sarcopenia defined by prediction equations and risk factors for CVD or osteoporosis. Against this backdrop, this study aimed to develop and cross-validate new prediction equations for estimating ASM, and to examine the relationship between sarcopenia defined by the prediction equations and risk factors for CVD or osteoporosis in Japanese men and women.

## Methods

### Subjects

Subjects were healthy Japanese men (*n* = 376) and women (*n* = 1025) aged 20–90 years, who were recruited from the community around the National Institute of Health and Nutrition (Tokyo, Japan) and randomly allocated to the following two groups: the development group (D group; 257 men, 913 women) and the cross-validation group (V group; 119 men, 112 women). This study was conducted as part of the Nutrition and Exercise Intervention Study (NEXIS) [24].

All subjects were sedentary or moderately active non-athletes who participated in swimming, stretching, or a “healthy gymnastics” program, but not in any vigorous sports activities. Men and women with CVD and stroke, as determined by a medical history questionnaire, were excluded from the study. Subjects did not take any medications, such as beta-blockers, steroids, or hormone replacement therapy. The purpose, procedures, and risks of the study were explained to all subjects prior to inclusion, and all subjects provided written informed consent before enrolling in the study. The study was performed in accordance with the guidelines of the Declaration of Helsinki and was approved by the Human Research Committee of the National Institute of Health and Nutrition, Tokyo, Japan (KENEI14-02). Body weight and height were recorded, and body mass index (BMI) was calculated as weight (kg) divided by height squared (m^2^). Waist circumference was measured at the superior border of the iliac crest.

### Analysis of blood samples

Blood was drawn from subjects in the seated position. Fasting (>12 h) blood samples were collected by venipuncture in tubes with or without ethylene diamine tetraacetic acid (for plasma or serum). Blood samples were centrifuged at 1500 rpm for 15 min and were stored at −20 °C. Serum concentration of triglycerides (TG) was determined using commercial kits (Mitsubishi Chemical Medience, Tokyo, Japan). Serum high-density lipoprotein cholesterol (HDL-C) was measured by an enzymatic method (Mitsubishi Chemical Medience). Fasting plasma glucose (FPG) was measured by the glucose dehydrogenase method. Whole-blood glycohemoglobin A1c (HbA1c) was measured by an enzymatic method (Glycohemoglobin A1c kit; Mitsubishi Chemical Medience).

### Analysis of arterial blood pressure at rest

Systolic blood pressure (SBP) and diastolic blood pressure (DBP) were measured at rest using a vascular testing device (Colin Medical Technology, Tokyo, Japan). Brachial-ankle pulse wave velocity (baPWV), which provides qualitatively similar information to that derived from central arterial stiffness, was measured by the volume plethysmographic method.

### Measures of whole-body DXA

Lean soft tissue mass and bone mineral density (BMD) were determined by whole-body DXA (Hologic QDR-4500A scanner; Hologic, Waltham, MA, USA). Whole-body lean soft tissue mass was divided into several regions, i.e., the arms, legs, and trunk. Body composition was determined by Hologic software version 11.2:3 for Windows (Hologic, Waltham, MA, USA). Reference values (ASM/height^2^) for class 1 and class 2 sarcopenia in each sex were defined as values one and two standard deviations (SD) below the sex-specific means of reference data for young adults aged 18–40 years, respectively. This study used reference values of height-adjusted ASM proposed by Sanada et al. [7]: 7.77 and 6.87 kg/m^2^ for men, and 6.12 and 5.46 kg/m^2^ for women, for class 1 and class 2 sarcopenia, respectively.

### Measures of fitness

Handgrip strength of the right upper limb was measured using a handheld dynamometer. In the standing position, with the arm straight by the side, subjects gripped the dynamometer as hard as possible for 3 s without pressing the instrument against the body or bending at the elbow. The value was recorded as the average of two trials. Leg extension power (LEP) was measured with the isokinetic leg power system (Anaero Press 3500; Combi Wellness, Tokyo, Japan) in the sitting position.

### Statistical analysis

All measurements and calculated values are expressed as mean ± SD. To develop prediction equations, stepwise multiple regression analyses were performed using data obtained from the D group, with DXA-measured ASM as a dependent variable and five easily obtainable measures (age, height, weight, waist circumference, and handgrip strength) as independent variables. The developed prediction equations were then used to calculate predicted ASM. Paired *t* tests were performed to determine differences between predicted ASM and DXA-measured ASM in the V group. We also compared the prediction equations for estimating ASM developed in the present study (using data from the V group) with those described in previous studies. Regression analysis between DXA-measured ASM and predicted ASM and Bland-Altman analysis was performed to validate the developed prediction equations in the V group. We compared mean values of physical characteristics, body composition, fitness, and risk factors for CVD between normal subjects and those with class 1 and class 2 sarcopenia by one-way analysis of covariance (ANCOVA) after adjusting for age. Multiple comparison was used as the post hoc test. The alpha level for testing significance was set at *P* < 0.05. All statistical analyses were performed using StatView version 5.0 for Windows (SAS Institute, Cary, NC, USA).

## Results

**Fig. 1:**
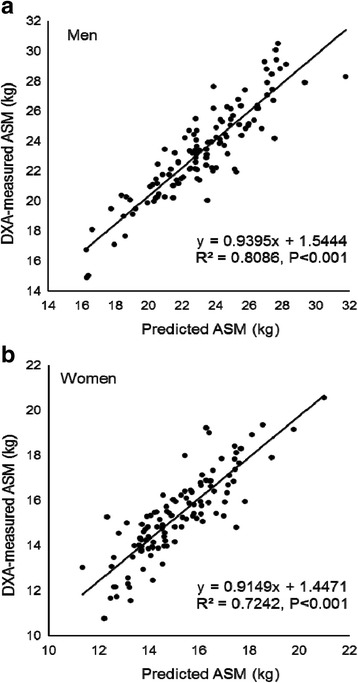
Correlation between DXA-measured ASM and predicted ASM in the cross-validation group for men (**a**) and women (**b**)

**Fig. 2:**
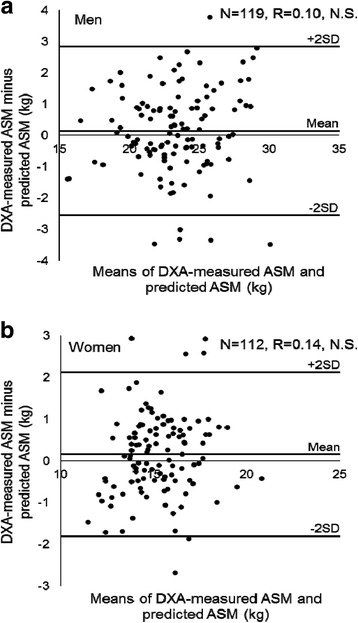
Bland-Altman plot of DXA-measured ASM and predicted ASM in the cross-validation group for men (**a**) and women (**b**)

The characteristics of subjects in the D and V groups are shown in Table 1. The prediction equations developed for ASM estimation are presented in Table 2. In both men and women, the prediction equations were highly correlated with DXA-measured ASM (*R* ^2^ = 0.88 and *R* ^2^ = 0.74, *P* < 0.001). When these equations were applied to the V group, a significant correlation was found between DXA-measured ASM and was predicted ASM in both men and women (*R* ^2^ = 0.81 and *R* ^2^ = 0.72, *P* < 0.001) (Fig. 1). “Difference between DXA-measured ASM and predicted ASM” was not significantly correlated with “means of DXA-measured ASM and predicted ASM” in both men and women (Fig 2). Bland-Altman analysis indicated no bias in the prediction of ASM for the V group.

**Table 1:** Subject characteristics

	Men			Women		
	Total	Development	Cross-validation	Total	Development	Cross-validation
*N*	376	257	119	1025	913	112
Age (years)	48 ± 17	49 ± 17	48 ± 16	54 ± 16	54 ± 16	53 ± 16
Height (cm)	169.9 ± 6.2	170.2 ± 6.1	169.5 ± 6.3	156.3 ± 6.1	156.3 ± 6.2	156.7 ± 5.9
Weight (kg)	67.7 ± 8.8	67.6 ± 9.0	67.7 ± 8.3	53.9 ± 7.3	53.9 ± 7.3	53.4 ± 7.4
BMI (kg/m^2^)	23.4 ± 2.6	23.3 ± 2.6	23.6 ± 2.5	22.0 ± 2.9	22.1 ± 2.9	21.7 ± 2.8
Waist circumference (cm)	83.1 ± 7.7	83.2 ± 8.1	83.0 ± 6.9	80.2 ± 9.4	80.2 ± 9.4	80.0 ± 9.3
% body fat (%)	20.0 ± 4.7	20.1 ± 4.7	19.7 ± 4.7	28.6 ± 5.8	28.7 ± 5.8	28.2 ± 5.7
Handgrip strength (kg)	40.2 ± 6.8	40.0 ± 6.6	40.6 ± 7.3	25.5 ± 4.5	25.4 ± 4.4	25.5 ± 4.8
ASM (kg)	23.2 ± 3.1	23.1 ± 3.1	23.4 ± 3.1	15.3 ± 2.0	15.3 ± 2.0	15.3 ± 1.9

Data are presented as mean ± SD. *BMI* body mass index, *ASM* appendicular skeletal muscle mass

**Table 2:** Prediction equations for ASM estimation based on multiple regression analysis of data obtained from the development group

	Prediction equations (kg)	*R* ^2^	SEE (kg)	*P* value
Men (*N* = 257)				
Step 1	ASM = 0.287 × weight (kg) + 3.681	0.685	1.75	< 0.001
Step 2	ASM = 0.460 × weight (kg)−0.251 × waist circumference (cm) + 12.867	0.864	1.15	< 0.001
Step 3	ASM = 0.408 × weight (kg)−0.209 × waist circumference (cm) + 0.072 × handgrip strength (kg) + 10.032	0.877	1.10	< 0.001
Women (*N* = 913)				
Step 1	ASM = 0.185 × weight (kg) + 5.330	0.472	1.43	< 0.001
Step 2	ASM = 0.155 × height (cm) + 0.138 × weight (kg)−16.291	0.674	1.13	< 0.001
Step 3	ASM = 0.121 × height (cm) + 0.128 × weight (kg) + 0.104 × handgrip strength (kg)−13.096	0.714	1.05	< 0.001
Step 4	ASM = 0.094 × height (cm) + 0.187 × weight (kg)−0.051 × waist circumference (cm) + 0.082 × handgrip strength (kg)−7.394	0.733	1.02	< 0.001
Step 5	ASM = 0.007 × age (years) + 0.095 × height (cm) + 0.196 × weight (kg)−0.061 × waist circumference (cm) + 0.087 × handgrip strength (kg)−7.896	0.735	1.02	< 0.001

*SEE* standard error of estimate

Table 3 shows the comparison of prediction equations for estimating ASM in the present (in the V group) and previous studies. The values of ASM estimated by our prediction equations were 23.2 ± 2.9 kg in men and 15.1 ± 1.7 kg in women; these values were not significantly different from DXA-measured ASM (23.4 ± 3.1 kg in men and 15.3 ± 1.9 kg in women). In men, however, the values of ASM estimated by the prediction equations of Baumgartner et al. (1998), Wen et al. (2011), Kulkarni et al. (2013), and Villani et al. (2014) were significantly lower than DXA-measured ASM. In women, the values of ASM estimated by the equations of Baumgartner et al. (1998), Wen et al. (2011), Visvanathan et al. (2012), and Kulkarni et al. (2013) were significantly lower, and that of Villani et al. (2014) significantly higher, than DXA-measured ASM. Our prediction equations had higher *R* ^2^ values than those developed previously in both men and women. In addition, our equations showed a lower standard error of estimation (SEE) than those of the previous studies.

**Table 3:** Comparison of prediction equations for estimating ASM in the present (in the cross-validation group) and previous studies

	ASM (kg)*	Difference from DXA-measured value (*P* value)	Correlation coefficients between DXA-measured and predicted ASM (*R* ^2^ value)	Significance of correlations(*P* value)	SEE (kg)
Men					
DXA-measured value	23.4 ± 3.1	–	–	–	–
Present study	23.2 ± 2.9	N.S.	0.81	< 0.001	1.10
Baumgartner RN et al. (1998)^a^	21.6 ± 2.0	< 0.001	0.71	< 0.001	1.58
Wen X et al. (2011)^b^	22.6 ± 2.3	< 0.001	0.75	< 0.001	1.63
Visvanathan R et al. (2012)^c^	23.3 ± 2.1	N.S.	0.68	< 0.001	1.95
Kulkarni B et al. (2013)^d^	21.7 ± 2.6	< 0.001	0.74	< 0.001	1.28
Villani AM et al. (2014)^e^	20.5 ± 2.2	< 0.001	0.59	< 0.001	1.90
Women					
DXA-measured value	15.3 ± 1.9	–	–	–	–
Present study	15.1 ± 1.7	N.S.	0.72	< 0.001	1.02
Baumgartner RN et al. (1998)^a^	14.1 ± 1.5	< 0.001	0.69	< 0.001	1.58
Tankó LB et al. (2002)^f^	15.1 ± 1.8	N.S.	0.54	< 0.001	1.70
Wen X et al. (2011)^b^	14.1 ± 1.9	< 0.001	0.65	< 0.001	1.63
Visvanathan R et al. (2012)^c^	14.2 ± 1.6	< 0.001	0.59	< 0.001	1.95
Kulkarni B et al. (2013)^d^	13.7 ± 1.7	< 0.001	0.66	< 0.001	1.05
Villani AM et al. (2014)^e^	16.4 ± 1.8	< 0.001	0.40	< 0.001	1.90

Mean ± SD. *SEE* standard error of estimate. Prediction equations of ASM for the present study were based on Table 2 (step 4 in men and step 5 in women). Prediction equations from previous studies using anthropometric measurements were as follows. ^a^ 0.2487 * weight (kg) + 0.0483 * height (cm) – 0.1584 * hip circumference (cm) + 0.0732 * handgrip strength (kg) + 2.5843 (only men) + 5.8828, ^b^ 0.193 * weight (kg) + 0.107 * height (cm) – 4.157 * gender (men = 1, women = 2) – 0.037 * age (years) – 2.631, ^c^ 10.047427 + 0.353307 * weight (kg) – 0.621112 * BMI – 0.022741 * age (years) + 5.096201, ^d^ 0.2 * weight (kg) + 0.14 * height (cm) –13.432 – 0.0445 * age (years) in men and 0.17 * weight (kg) + 0.102 * height (cm) – 9.852 – 0.028 * age (years) in women, ^e^ 16.77–0.036 * age (years) + 0.385 * weight (kg) – 0.873 * BMI, ^f^ 0.11 * weight (kg) –13.3 – 0.05 * age (years) + 16.1 * height (m)

Health-related indices for men in the D and V groups are shown in Table 4. When sarcopenia was defined by DXA-measured ASM, weight, BMI, and waist circumference in subjects with class 1 and class 2 sarcopenia were significantly lower compared to normal subjects (*P* < 0.05). The same results were obtained when sarcopenia was defined by estimated ASM. LEP/weight, handgrip strength, whole-body BMD, arm BMD, and leg BMD in subjects with class 1 and 2 sarcopenia according to both definitions (i.e., DXA-measured ASM and predicted ASM) were significantly lower compared to normal subjects (*P* < 0.05). baPWV was significantly higher in subjects with class 1 and 2 sarcopenia according to both definitions than in normal subjects (*P* < 0.05).

**Table 4:** Health-related indices for men with sarcopenia as defined by DXA-measured ASM or predicted ASM in the development and cross-validation groups

	Sarcopenia defined by DXA-measured ASM			*P* value*		Sarcopenia defined by predicted ASM			*P* value*	
	Normal	Class 1 sarcopenia	Class 2 sarcopenia	One-way ANCOVA	Post hoc analysis	Normal	Class 1 sarcopenia	Class 2 sarcopenia	One-way ANCOVA	Post hoc analysis
*N*	230 (61.2%)	118 (31.4%)	28 (7.4%)	–	–	234 (62.2%)	122 (32.5%)	20 (5.3%)	–	–
Age (years)	45 ± 14	51 ± 18	62 ± 22	–	–	45 ± 14	52 ± 18	66 ± 21	–	–
Height (cm)	170.7 ± 6.1	169.0 ± 6.2	168.0 ± 5.7	N.S.		170.5 ± 6.1	169.6 ± 6.0	166.1 ± 6.2	N.S.	
Weight (kg)	71.1 ± 8.1	63.3 ± 6.2	57.1 ± 6.7	< 0.001	a, b, c	71.4 ± 8.0	62.7 ± 5.7	54.5 ± 4.7	< 0.001	a, b, c
BMI (kg/m^2^)	24.4 ± 2.4	22.2 ± 1.7	20.2 ± 1.9	< 0.001	a, b, c	24.5 ± 2.3	21.8 ± 1.6	19.8 ± 1.4	< 0.001	a, b, c
Waist circumference (cm)	84.7 ± 7.7	81.0 ± 6.8	78.9 ± 7.7	< 0.001	a, b	84.8 ± 7.6	80.8 ± 7.1	78.1 ± 6.3	< 0.001	a, b
% body fat (%)	19.7 ± 4.7	20.3 ± 4.6	20.7 ± 4.4	N.S.		20.4 ± 4.7	19.4 ± 4.7	18.7 ± 4.3	< 0.001	a
LEP/weight (W/kg)	24.5 ± 6.2	20.0 ± 5.8	18.1 ± 5.1	< 0.001	a, b	24.2 ± 6.3	20.8 ± 5.9	15.9 ± 4.8	< 0.001	a, b, c
Handgrip strength (kg)	42.6 ± 6.2	37.3 ± 5.6	33.1 ± 5.9	< 0.001	a, b, c	42.7 ±6.2	37.1 ± 5.0	29.5 ± 4.3	< 0.001	a, b, c
Whole-body BMD (g/cm^2^)	1.173 ± 0.108	1.118 ± 0.106	1.052 ± 0.098	< 0.001	a, b, c	1.176 ± 0.110	1.109 ± 0.095	1.040 ± 0.110	< 0.001	a, b, c
Arm BMD (g/cm^2^)	0.818 ± 0.065	0.770 ± 0.053	0.735 ± 0.051	< 0.001	a, b, c	0.818 ± 0.065	0.770 ± 0.053	0.725 ± 0.060	< 0.001	a, b, c
Lumbar spine BMD (g/cm^2^)	1.055 ± 0.171	1.042 ± 0.159	0.975 ± 0.186	< 0.05	b	1.061 ± 0.169	1.032 ± 0.164	0.941 ± 0.171	< 0.01	b, c
Leg BMD (g/cm^2^)	1.282 ± 0.111	1.204 ± 0.107	1.123 ± 0.095	<0.001	a, b, c	1.281 ± 0.111	1.198 ± 0.104	1.119 ± 0.119	< 0.001	a, b, c
FPG (mg/dl)	92.3 ± 10.2	90.0 ± 10.3	90.1 ± 11.6	< 0.001	a	92.3 ± 10.0	89.8 ± 10.2	91.3 ± 13.9	< 0.001	a
HbA1c (%)	5.23 ± 0.45	5.23 ± 0.43	5.52 ± 0.52	N.S.		5.23 ± 0.44	5.24 ± 0.46	5.54 ± 0.45	N.S.	
TG (mg/dl)	105.0 ± 56.2	98.1 ± 55.1	102.7 ± 59.6	N.S.		105.6 ± 57.3	95.3 ± 50.8	113.2 ± 68.5	N.S.	
HDL-C (mg/dl)	56.0 ± 12.7	58.2 ± 12.6	58.3 ± 14.4	N.S.		56.2 ± 13.2	57.3 ± 11.7	62.2 ± 14.2	N.S.	
SBP (mmHg)	122 ± 14	121 ± 15	129 ± 18	< 0.05	b, c	122 ± 14	122 ± 14	129 ± 20	N.S.	
DBP (mmHg)	75 ± 11	75 ± 11	74 ± 10	< 0.001	N.S.	75 ± 11	75 ± 10	71 ± 10	< 0.001	N.S.
baPWV (cm/s)	1285 ± 214	1378 ± 291	1609 ± 408	< 0.01	a, b, c	1291 ± 222	1378 ± 300	1653 ± 380	< 0.05	a, b, c
DXA-measured ASM (kg)	24.8 ± 2.5	21.2 ± 1.7	18.5 ± 1.6	< 0.001	a, b, c	24.5 ± 2.7	21.5 ± 2.1	18.4 ± 2.1	< 0.001	a, b, c
Predicted ASM (kg)	24.4 ± 2.6	21.6 ± 2.0	19.2 ± 2.1	< 0.001	a, b, c	24.5 ± 2.5	21.4 ± 1.7	18.1 ± 1.6	< 0.001	a, b, c

Data are presented as mean ± SD. *One-way ANCOVA with adjustment for the covariate of age and post hoc analysis using the least significant *t* test (mean difference between two groups): *a* normal vs. class 1, *b* normal vs. class 2, *c* class 1 vs. class 2, all *P* < 0.05. *BMI* body mass index, *LEP* leg extension power, *BMD* bone mineral density, *FPG* fasting plasma glucose, *HbA1c* glycohemoglobin A1c, *TG* triglycerides, *HDL-C* high-density lipoprotein cholesterol, SBP systolic blood pressure, *DBP* diastolic blood pressure, *baPWV* brachial-ankle pulse wave velocity, *ASM* appendicular skeletal muscle mass

Health-related indices for women in the D and V groups are shown in Table 5. Weight, BMI, waist circumference, handgrip strength, whole-body BMD, and regional BMD in subjects with classes 1 and 2 sarcopenia according to both definitions were significantly lower compared to normal subjects (*P* < 0.05). baPWV was significantly higher in subjects with class 2 sarcopenia according to both definitions compared to normal subjects (*P* < 0.05).

**Table 5:** Health-related indices for women with sarcopenia as defined by DXA-measured ASM or predicted ASM in the development and cross-validation groups

	Sarcopenia defined by DXA-measured ASM			*P* value*		Sarcopenia defined by predicted ASM			*P* value*	
	Normal	Class 1 sarcopenia	Class 2 sarcopenia	One-way ANCOVA	Post hoc analysis	Normal	Class 1 sarcopenia	Class 2 sarcopenia	One-way ANCOVA	Post hoc analysis
*N*	577 (56.3%)	358 (34.9%)	90 (8.8%)	–	–	557 (54.3%)	443 (43.2%)	25 (2.4%)	–	–
Age (years)	54 ± 16	54 ± 16	56 ± 16	–	–	52 ± 15	56 ± 17	64 ± 19	–	–
Height (cm)	156.4 ± 6.3	156.5 ± 5.9	155.8 ± 6.0	N.S.		157.0 ± 6.0	155.8 ± 6.1	151.0 ± 5.5	< 0.001	a, b, c
Weight (kg)	56.9 ± 7.2	50.7 ± 5.3	47.0 ± 4.9	< 0.001	a, b, c	58.2 ± 6.4	49.2 ± 4.1	40.8 ± 3.5	< 0.001	a, b, c
BMI (kg/m^2^)	23.3 ± 2.8	20.7 ± 2.0	19.4 ± 1.8	< 0.001	a, b, c	23.6 ± 2.6	20.3 ± 1.7	17.9 ± 1.4	< 0.001	a, b, c
Waist circumference (cm)	82.6 ± 9.7	77.5 ± 8.0	75.0 ± 7.4	< 0.001	a, b, c	83.0 ± 9.7	77.0 ± 7.7	72.3 ± 7.7	< 0.001	a, b, c
% body fat (%)	28.8 ± 6.1	28.2 ± 5.4	29.0 ± 4.7	N.S.		30.3 ± 5.7	26.7 ± 5.1	23.8 ± 5.1	< 0.001	a, b, c
LEP/weight (W/kg)	15.4 ± 4.2	15.0 ± 4.2	13.6 ± 3.6	< 0.001	b, c	15.4 ±4.2	14.8 ± 4.2	12.5 ± 3.9	N.S.	
Handgrip strength (kg)	26.3 ± 4.7	24.7 ± 3.8	22.7 ± 4.0	< 0.001	a, b, c	27.2 ± 4.3	23.6 ± 3.6	19.1 ± 2.5	< 0.001	a, b, c
Whole-body BMD (g/cm^2^)	1.031 ± 0.122	1.004 ± 0.108	0.972 ± 0.112	< 0.001	a, b, c	1.044 ± 0.114	0.986 ± 0.112	0.919 ± 0.116	< 0.001	a, b, c
Arm BMD (g/cm^2^)	0.652 ± 0.067	0.633 ± 0.065	0.614 ± 0.064	< 0.001	a, b, c	0.659 ± 0.062	0.624 ± 0.066	0.581 ± 0.079	< 0.001	a, b, c
Lumbar spine BMD (g/cm^2^)	0.992 ± 0.171	0.942 ± 0.158	0.901 ± 0.169	< 0.001	a, b, c	1.005 ± 0.165	0.928 ± 0.161	0.810 ± 0.149	< 0.001	a, b, c
Leg BMD (g/cm^2^)	1.060 ± 0.110	1.018 ± 0.102	0.972 ± 0.094	< 0.001	a, b, c	1.070 ± 0.100	1.005 ±0.105	0.916 ± 0.112	< 0.001	a, b, c
FPG (mg/dl)	90.1 ± 10.0	87.9 ± 9.4	87.7 ± 11.4	< 0.01	a, b	90.3 ± 10.1	88.0 ± 9.8	82.9 ± 6.6	< 0.001	a, b, c
HbA1c (%)	5.40 ± 0.43	5.35 ± 0.40	5.33 ± 0.40	< 0.05	N.S.	5.37 ± 0.43	5.38 ± 0.40	5.37 ± 0.36	N.S.	
TG (mg/dl)	85.5 ± 47.2	81.5 ± 40.8	77.7 ± 35.9	N.S.		88.9 ± 49.9	77.4 ± 35.7	68.4 ± 24.2	< 0.001	a, b
HDL-C (mg/dl)	68.3 ± 15.5	70.7 ± 15.7	69.0 ± 15.6	N.S.		66.7 ± 15.1	71.8 ± 15.8	78.4 ± 13.6	< 0.001	a, b, c
SBP (mmHg)	120 ± 17	116 ± 17	119 ± 20	< 0.001	a, c	120 ± 17	117 ± 17	124 ± 26	< 0.001	a,c
DBP (mmHg)	71 ± 11	68 ± 10	70 ± 11	< 0.001	a	71 ± 11	68 ± 10	69 ± 13	< 0.001	a
baPWV (cm/s)	1282 ± 237	1296 ± 257	1385 ± 336	< 0.001	b, c	1267 ± 226	1320 ± 272	1503 ± 413	< 0.01	a, b, c
DXA-measured ASM (kg)	16.3 ± 1.8	14.3 ± 1.1	12.7 ± 1.1	< 0.001	a, b, c	16.2 ± 1.8	14.4 ± 1.5	12.3 ± 1.1	< 0.001	a, b, c
Predicted ASM (kg)	15.7 ± 1.7	14.7 ± 1.4	13.9 ± 1.4	< 0.001	a, b, c	16.1 ± 1.5	14.3 ± 1.2	12.1 ± 1.0	< 0.001	a, b, c

Data are presented as mean ± SD. *One-way ANCOVA with adjustment for the covariate of age and post hoc analysis using the least significant *t* test (mean difference between two groups): *a* normal vs. class 1, *b* normal vs. class 2, *c* class 1 vs. class 2, all *P* < 0.05. *BMI* body mass index, *LEP* leg extension power, *BMD* bone mineral density, *FPG* fasting plasma glucose, *Hb1c* glycohemoglobin A1c, *TG* triglycerides, *HDL-C* high-density lipoprotein cholesterol, *SBP* systolic blood pressure, *DBP* diastolic blood pressure, *baPWV* brachial-ankle pulse wave velocity, *ASM* appendicular skeletal muscle mass

## Discussion

Population-specific prediction equations for estimating ASM have been reported previously. However, no prediction equations have been developed in Japanese populations, and no study has examined the relationship between sarcopenia defined by prediction equations and risk factors for CVD or osteoporosis. The comparison of our prediction equations with those developed in previous studies led to the following key findings: (1) our prediction equations were more applicable to Japanese populations than those developed in previous studies; and (2) sarcopenia defined by predicted ASM was related to risk factors for osteoporosis and CVD, as well as sarcopenia defined by DXA-measured ASM.

Several studies have reported prediction equations for ASM assessment using anthropometric measurements in other countries. Prediction equations proposed by Baumgartner et al., Wen et al., Visvanathan et al., Kulkarni et al., and Villani et al. showed high correlations (*R* ^2^ = 0.73–0.93), with SEEs of 0.94–1.95 kg [4, 20–23]. On the other hand, the prediction equations developed in our study showed a higher correlation in both men (*R* ^2^ = 0.88) and women (*R* ^2^ = 0.74) (Table 2), with a low SEE of 1.10 kg in men and 1.02 kg in women, which were consistent with previous studies. Moreover, our prediction equations also showed a high correlation in men (*R* ^2^ = 0.81) and women (*R* ^2^ = 0.72) in the V group (Fig. 1). No significant difference was observed between DXA-measured ASM and predicted ASM estimated by the prediction equations in the V group (Table 3). These results suggest that our prediction equations have high accuracy for estimating ASM in a Japanese population.

We also examined whether previously reported prediction equations were applicable in a Japanese population. In both men and women, the values of predicted ASM estimated by our prediction equations were more highly correlated with DXA-measured ASM compared to the values of predicted ASM estimated by previously reported prediction equations in the V group (Table 3). Previously reported prediction equations have shown high correlations in previous studies (*R* ^2^ = 0.73–0.93), but correlations were lower in subjects who participated in the present study (*R* ^2^ = 0.40–0.75). Moreover, the values of ASM estimated by previously reported equations, except for those of Visvanathan et al. (in men) and Tankó et al. (in women), were significantly different from the values of DXA-measured ASM. Although the values of ASM estimated by the prediction equations of Visvanathan et al. and Tankó et al. did not significantly differ from DXA-measured ASM, *R* ^2^ values of these studies between predicted and DXA-measured ASM were relatively low. Subjects of these previous studies were Hispanic, non-Hispanic, Caucasian, Chinese, Indian, and Australian. However, most prediction values of ASM from previous studies have underestimated ASM compared to those in the present study, and it is not likely showed ethnical difference in those predicted ASM values. These findings suggest that previously reported prediction equations may not be applicable in Japanese populations.

According to our prediction equations, waist circumference negatively affected ASM in both men and women (Table 2). A negative correlation between waist circumference and skeletal muscle mass has been reported in previous studies [25, 26]. For instance, Roubenoff showed that an increase in fat mass, especially visceral fat mass, elevated inflammatory cytokines, which in turn accelerated muscle catabolism and contributed to initiating and sustaining sarcopenic obesity [27]. Moreover, Schrager et al. reported that sarcopenic obesity was associated with elevated levels of IL-6, C-reactive protein, and soluble IL-6 receptor and that central obesity was more proinflammatory than generic obesity [28]. In the present study, waist circumference, which was selected as a predictor variable of ASM, negatively affected ASM, as reported by previous studies.

To examine the clinical significance of the prediction equations developed in the present study, we divided subjects into normal, class 1 sarcopenia, and class 2 sarcopenia groups according to DXA-measured ASM and predicted ASM estimated by these prediction equations. Whole-body BMD, arm BMD, and leg BMD in subjects with classes 1 and 2 sarcopenia according to both definitions (i.e., DXA-measured ASM and predicted ASM) were significantly lower compared to normal subjects for men and women (Tables 4 and 5). Moreover, baPWV was significantly higher in subjects with class 2 sarcopenia according to both definitions compared to normal subjects for men and women (Tables 4 and 5). In addition, baPWV was significantly higher in subjects with class 2 sarcopenia according to both definitions than in normal subjects for men and women (Tables 4 and 5). baPWV has been widely used as a non-invasive marker to evaluate arterial stiffness, but there is little evidence for its prognostic value in the general population. Takashima et al. reported that higher baPWV is an independent predictor of future CVD events in the general Japanese population [29], suggesting that higher baPWV in subjects with sarcopenia in the present study may be associated with CVD risk factors. On the other hand, HbA1c did not significantly differ between subjects with sarcopenia and normal subjects in both men and women (Tables 4 and 5). As discussed earlier, we previously reported that although sarcopenia is associated with thin body mass, it is associated with more glycation of serum proteins in Japanese adult men, independently of waist circumference [7]. Therefore, DXA-measured ASM may be more sensitive in detecting the risk of diabetes (e.g., glycation of serum proteins) compared to the prediction equations for estimating ASM developed in this study.

One limitation of this study was the inability to evaluate the effect of menopause status on the prediction equations, given the lack of information on menopause status for most female subjects.

## Conclusions

We developed and cross-validated novel prediction equations in Japanese men and women. Our analyses revealed that the prediction equations developed in previous studies are not adequate for Japanese populations, and that our novel prediction equations have validated clinical significance.
